# Evaluating the care received by patients with oesophago-gastric (OG) cancer: the richer picture provided by linked datasets

**DOI:** 10.23889/ijpds.v1i1.341

**Published:** 2017-04-19

**Authors:** Christian Brand, Mira Varagunam, Georgina Chadwick, David Cromwell

**Affiliations:** 1 Clinical Effectiveness Unit, Royal College of Surgeons of England

## Objectives

The national oesophago-gastric cancer audit evaluates the care given to patients with OG cancer diagnosed in England and Wales, identifying areas for local services to improve and so achieve better patient outcomes. While the audit follows a standard approach by collecting a core dataset, it extends this by linking to other national databases in order to (1) reduce the burden of data collection, (2) add data that could not otherwise be obtained, (3) verify the quality of audit data. This presentation will provide an overview of the modern approach to national clinical audits and introduce anticipated future developments. 

## Approach

The data collected by hospitals and submitted to the audit is expanded by linkage to radiotherapy data (RTDS), date of death from the Office for National Statistics and administrative hospital admission data (HES). The linked data enables the Audit to: (1) examine whether radiotherapy regimens used in definitive oncology comply with guideline recommendations, using dosage information drawn from RTDS; (2) publish comparative short-term survival figures for NHS trusts and surgeons using ONS date of death; and (3) assess the quality of data submitted from hospitals by comparing coded procedural information in HES and the audit dataset.

## Results

The analysis of the linked data has led to several important audit findings, namely: (1) Access to RTDS data confirmed reasonable compliance with official guidelines on recommended radiotherapy dosing schedules. (2) Linked mortality data has provided robust and credible estimates of surgical outcomes and is a critical output to NHS trusts and surgeons, as well as the wider public. Our analysis has highlighted a gradual decline in post-operative mortality. (3) Linkage of audit and HES data identified gaps in the recording of endoscopic stenting, with many procedures not being submitted by participating trusts. These diverse results underline the usefulness of linked data in revealing a more complete picture of the care received by OG cancer patients.

## Conclusion

Linked data has become integral for the successful implementation of a national clinical audit, supplementing the core dataset with information that would otherwise be unavailable or difficult and costly to collect. Linkage enhances the audit's ability to assess health services' compliance with professional standards and to give service providers the opportunity to benchmark their performance. Future developments are expected to expand on this by including primary care service use before diagnosis.

